# A comprehensive data set of physical and human-dimensional attributes for China’s lake basins

**DOI:** 10.1038/s41597-022-01649-z

**Published:** 2022-08-25

**Authors:** Tan Chen, Chunqiao Song, Chenyu Fan, Jian Cheng, Xuejun Duan, Lei Wang, Kai Liu, Shulin Deng, Yue Che

**Affiliations:** 1 Key Laboratory of Watershed Geographic Sciences, Nanjing Institute of Geography and Limnology, Chinese Academy of Sciences, Nanjing, 210008 China; 2 College of Resources and Environment, University of Chinese Academy of Sciences, Beijing, 100049 China; 3 School of Geography and Ocean Science, Nanjing University, Nanjing, 210023 China; 4 School of Geography and Planning, Nanning Normal University, Nanning, 530001 China; 5 Shanghai Key Lab for Urban Ecological Processes and Eco-Restoration, School of Ecological and Environmental Sciences, East China Normal University, Shanghai, 200241 China

**Keywords:** Hydrology, Geography

## Abstract

Lakes provide water-related ecosystem services that support human life and production. Nevertheless, climate changes and anthropogenic interventions remarkably altered lake and basin hydrology in recent decades, which pose a significant threat to lacustrine ecosystems. Therefore, assessments of lacustrine ecosystems require the spatial and temporal characteristics of key physical and human-dimensional attributes for lakes and lake basins. To facilitate stakeholders obtaining comprehensive data of lake basins in China, we compiled the comprehensive data set for China’s lake basins (CODCLAB) mostly from publicly available data sources based on spatial analysis and mathematical statistics methods in this study. The CODCLAB is available in three data formats, including raster layers (Level 1) in “tiff” format, vector shapefiles (Level 2), and attribute tables (Level 3). It covers 767 lakes (>10 km^2^) in China and their basin extent associating with 34 variables organized into five categories: Hydrology, Topography, Climate, Anthropogenic, and Soils. This unique database will provide basic data for research on the physical processes and socioeconomic activities related to these lakes and their basins in China and expect to feed a broad user community for their application in different areas.

## Abstract (summary)

**Table:** 

Measurement(s)	Hydrology, Topography, Climate, Anthropogenic, and Soils for China’s lake basins
Technology Type(s)	spatial analysis and mathematical statistics
Factor Type(s)	CODCLAB_Level1 • CODCLAB_Level2 • CODCLAB_Level3
Sample Characteristic - Location	China’s lake basins

## Background & Summary

Lakes are increasingly influenced by anthropogenic pressures and environmental changes (e.g., changing climate) that can modify their hydrology and ecological functions^1,2^. A growing body of literature has evidenced that it is essential to know how lakes respond to natural and anthropogenic factors^3–6^. These evidence consistently indicates that intensified driving forces have been weakening the environmental, economic, and public health benefits provided by lakes^7^. For instance, land use changes (e.g., reclamation projects, irrigated agriculture) in the lake basin can modify lake hydrologic regimes beyond natural ranges. While environmental changes (e.g., changing climate or soil geology) may accelerate human pressure on lake hydrology^8,9^. Yet, the interaction between lakes and the environment is very complex. Concurrently, the lake dynamics can indicate the course of their basin changes, and the basin changes can affect the properties of lakes in reverse^10^. Researchers and policymakers are trying to apply effective solutions to alleviate climate variability and human footprints on lakes^11,12^, which necessitates large amounts of data related to these physical and anthropogenic processes herein^1,13^. Therefore, for a comprehensive knowledge about the changes occurred in lakes or lacustrine ecosystems often necessitate more background information on the spatial-temporal characteristics of key attributes at the basin scale that users are interested in, such as topography, climate, anthropogenic, etc.

Hydrological data of lakes in the regional or global scale are increasingly generated and applied in recent years, such as lake area, level, and volume data from the ground- and satellite-based observations^6,14^. HydroLAKES was arguably one of the most prominent choices and was widely applied in limnologic and hydrologic studies. The HydroLAKES database distinguished 1.42 million lakes with an area above 0.1 km^2^ and provided their vector boundaries associated with basic attributes^15^. However, researchers rarely paid attention to comprehensive hydrological, physical, and cultural characteristics at the basin scale of lakes. As a pioneer in comprehensive basin-scale data sets, the HydroATLAS database offered hydro-environmental sub-basin and river characteristics globally, accompanied by 56 variables in six categories^16^. Although the HydroATLAS database is valuable for basin-scale studies with fully global data references, the comprehensive attributes provided by HydroATLAS are not well applicable to China’s lake basins due to the lack of enough local validations. For the lake basins in China, there is no HydroATLAS-like comprehensive watershed data set well constrained by local data quality control. Instead, Chinese scholars pay more attention to the dynamics of lakes and basins in key areas (e.g., Tibetan Plateau, and Yangtze River basin)^17–21^, as well as the characteristics of various attributes based on sample points at the national scale^22,23^. Despite these advancements, users are more willing to select the data from a set of basin-scale characteristic data sets consistently.

**Fig. 1:**
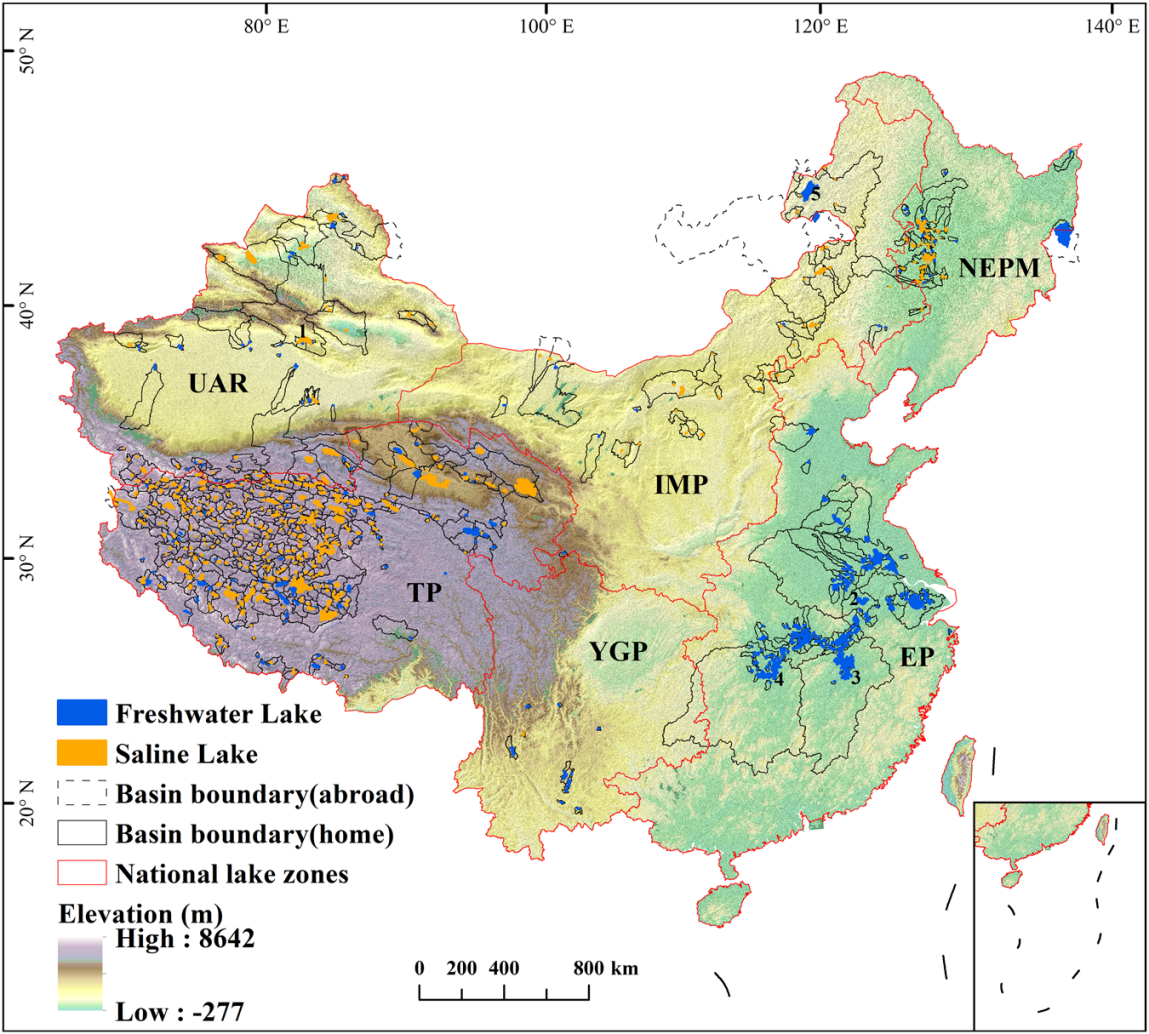
Map of China lakes and basins included in the generated data set. National lake zones (Fig. S1, Table S1) include Yunnan-Guizhou Plateau (YGP), Tibetan Plateau (TP), Uygur Autonomous Region (UAR), Inner Mongolia Plateau (IMP), Northeast Plains and Mountains (NEPM), and Eastern Plains (EP). Five large lakes with the sub-basins in CODCLAB include 1 Bosten Lake, 2 Chaohu Lake, 3 Poyang Lake, 4 Doting Lake, and 5 Hulun Lake.

To facilitate stakeholders obtaining comprehensive data of lake basins in China, we introduce the comprehensive dataset for China’s lake basins (CODCLAB). We provided 767 Chinese lakes (≥10 km^2^) and their basin boundaries with geographic reference in the CODCLAB dataset, in which the study lakes and their basins represent nearly 93% of the total lake area and 36% of the land area in China, respectively (Fig. 1). In addition, CODCLAB also provided extensive variables at basin scale that are organized into five categories (Hydrology, Topography, Climate, Anthropogenic, and Soils) based on publicly available data sources (Table 1).

**Table 1:** Spatiotemporal variables of CODCLAB.

Category	Variable	Source data	Spatial resolution (G:raster,V:vector)	Temporal resolution (S:static,D:interval)	Source year	Reference/Source
Hydrology	Lake extent	JRC GSW	G: 30 m	D: unequal	1984-2020	Pekel, *et al*.^24^
	Lake volume	HydroLakes	V: ~1:250,000	S	most recent	Messager, *et al*.^15^
	Residence time	HydroLakes	V: ~1:250,000	S	most recent	Messager, *et al*.^15^
	Watershed area	HydroSheds	V: ~1:250,000	S	most recent	Linke, *et al*.^16^
Topography	Elevation	SRTM1 DEM	G: 30 m	S	2000	USGS
	Terrain slope	SRTM1 DEM	G: 30 m	S	2000	USGS
	Relief amplitude	SRTM1 DEM	G: 30 m	S	2000	USGS
Climate	Temperature	Meteorological stations	G: 1 km	D: yearly	1980-2015	RESDC
	Precipitation	Meteorological stations	G: 1 km	D: yearly	1980-2015	RESDC
	Evapotranspiration	China terrestrial evapotranspiration	G: 0.1°	D: monthly	1982-2017	Ma, *et al*.^42^
	Pressure	CMFD	G: 0.1°	D: yearly	1979-2018	He, *et al*.^43^
	Specific humidity	CMFD	G: 0.1°	D: yearly	1979-2018	He, *et al*.^43^
	Wind speed	CMFD	G: 0.1°	D: yearly	1979-2018	He, *et al*.^43^
	2m-air temperature	CMFD	G: 0.1°	D: yearly	1979-2018	He, *et al*.^43^
	Precipitation rate	CMFD	G: 0.1°	D: yearly	1979-2018	He, *et al*.^43^
Anthropogenic	Population count	China population	G: 1 km	D: 5 years	1990-2015	RESDC
	Population density	WorldPop	G: 1 km	D: yearly	2000-2020	Tatem^44^
	Nighttime lights	NPP-VIIRS-like NTL	G: 500 m	D: yearly	2000-2018	Chen, *et al*.^45^
	Human footprint	Wild V4	G: 1 km	S	1993,2009	Venter, *et al*.^46^
	Gross domestic product	China GDP	G: 1 km	D: 5 years	1995-2015	RESDC
	Land use/cover	CLCD	G: 30 m	D: yearly	1990-2019	Yang and Huang^47^
Soils	Soil property (×12)	National soil information grids	G: 100 m-1 km	D: unequal	2010-2018	Liu, *et al*.^48^
	Soil moisture	SMC_V3	G: 0.05°	D: monthly	2002.7-2018.12	Meng, *et al*.^49^

Our compiled CODCLAB dataset is expected to facilitate more users to access the spatial-temporal characteristics of key attributes for the lake basins of China and be applied in different areas. Further, CODCLAB can provide data reference for comprehensive evaluation of lake basins, mixing natural and human sciences. For example, the anthropogenic dataset of CODCLAB could be used to advance studies of anthropogenic effects on the lake environment. Moreover, the CODCLAB can also directly support the response of lake hydrology to climate change and various natural factors.

## Methods

### Data compilation

**Fig. 2:**
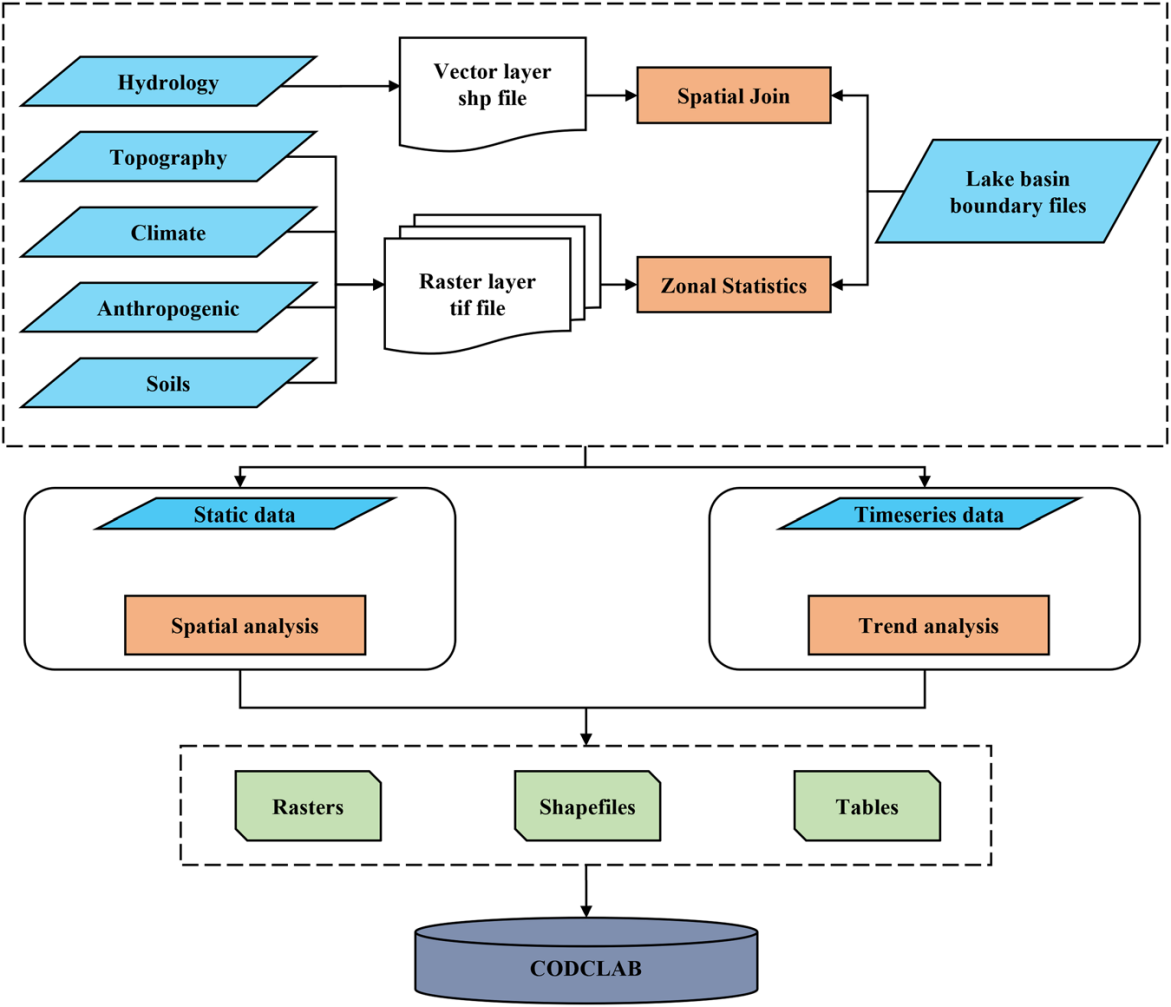
Flowchart for reconstructing the CODCLAB dataset.

We applied spatial analysis and mathematical statistics methods to compile the CODCLAB dataset (Fig. 2). The CODCLAB dataset is organized into five categories (Hydrology, Topography, Climate, Anthropogenic, and Soils) and contains 749 extended attributes (Table 2). First, the extended attributes within vector and raster files were correspondingly assigned the lake basins based on spatial join and zonal statics methods by Geographic Information System (GIS) tools, respectively. Then, the lake basin scaled static and time series data were processed to generate a final dataset including tables, shapefiles, and raster files.

### Lake and lake-basin delineation

1. Lake water extent delineation
  In this study, we detected the maximum water area of lakes (>10 km^2^) in China from 1984 to 2020 based on the Global Surface Water (GSW) datasets of the Joint Research Centre (JRC) (https://global-surface-water.appspot.com/). The JRC GSW dataset is a global waterbody data set with high temporal and spatial resolution and a long time sequence that was produced by an expert system of combining evidentiary reasoning and visual interpretation^24^. With high accuracy, the JRC GSW dataset has been widely used as a key hydro-science data source^25–27^.
  We used the Max Water Extent (MWE) data layer of the JRC GSW dataset in a version of 1.3 as the pending lake boundaries, reflecting the maximum inundation extent of global surface water from 1984 to 2020. Further, we removed the objects corresponding to other water bodies of non-natural lakes based on artificial interpretation methods one by one, such as rivers, artificial lakes (reservoirs), paddy fields and wetlands, etc. When removing the non-natural lakes, we referred to the google earth historical images, and basic geographic data, including the national basic geographic database of lake point data from the second National Lake Survey and other relevant literature^28,29^. Finally, the maximum water extent of 767 lakes in China from 1984 to 2020 was obtained. The study lakes (Fig. 1) include 298 freshwater lakes (39%) and 469 saline lakes (61%)^28,30,31^.
2. Lake-basin delineation

**Fig. 3:**
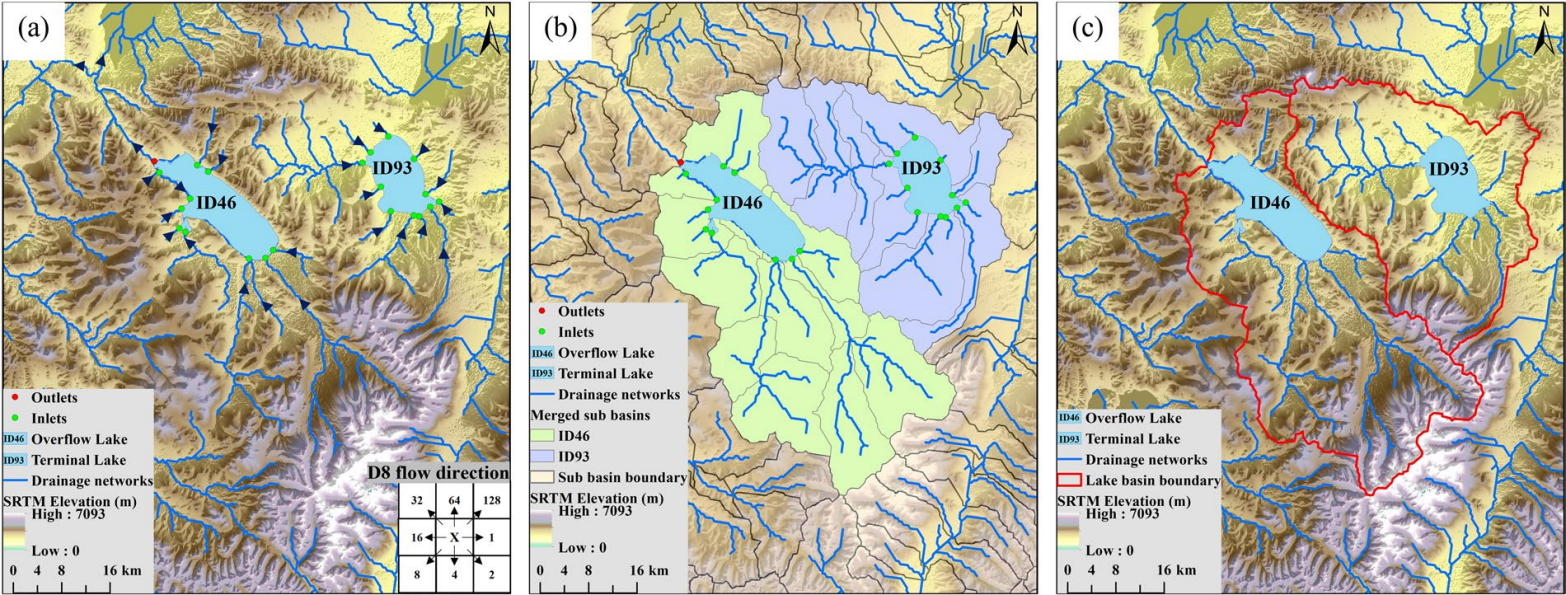
Diagram of lake basin delineation. (**a**) shows the determination of inlets and outlets of the lake based on flow direction calculated by the D8 algorithm. (**b**) shows the upstream drainage areas merged by all the sub-basins that flow into the lake. (**c**) are the basin delineation results of two example lakes: the overflow lake (ID46) and the terminal lake (ID93).

Based on HydroBASINS, HydroRIVERS, and Shuttle Radar Topography Mission (SRTM) Digital Elevation Model (DEM) datasets^32–34^, we delineated the basin boundary data for a total of 767 lakes (MWE > 10 km^2^) in China (Fig. 1). Figure 3 shows the lake basin delimitation process. Firstly, we computed the flow directions based on SRTM DEM according to the D8 algorithm^35^ (Fig. 3(a)). Then, we determinated the inlets, outlets, and sources of rivers of all lakes by overlaying the lake water extent with SRTM DEM and river works derived from HydroRIVERS (Fig. 3(a)). Secondly, we merged or edited the finer-level geometry of HydroBASINS, which contained all the rivers that flow through the lake (Fig. 3(b)). For five large lakes with broad watershed extents, we further delineated their secondary sub-basins with reference studies or maps. The five large lakes included Bosten Lake, Chaohu Lake, Poyang Lake, Dongting Lake, and Hulun Lake (Fig. 1). Thus, 767 lake basins and 805 sub-basins were delineated eventually.

### Processing of key attributes data by lake basin

1. Lake-basin attributes assignment
  This study assigned the CODCLAB attributes in both the vector and raster files one-to-one to the lake basins based on the spatial join and zonal statics methods from GIS tools, respectively (Fig. 2). The spatial join tool can join attributes from one feature to another based on the spatial relationship. The target features and the joined characteristics from the join features are written to the output feature class. Therefore, spatial join is suitable for lake-basin assignments like vector hydrologic attributes of CODCLAB. Further, the zonal statistics GIS tool can calculate statistics on values of a raster within the zones of another dataset. Therefore, according to the CODCLAB attributes of the raster data format, we used lake-basin boundaries to do zonal statistics for these attributes and realized the CODCLAB attributes assignment of lake basins based on raster files.
2. Attributes processing

#### Lake area extraction

JRC GSW water dynamic maps were used in the study to extract the lake area from 1984 to 2020. The GSW water dynamic maps (1984–2020) were created through automated process mining of the archive of the Landsat 7 ETM + and Landsat 8 OLI missions with a spatial resolution of 30 m^24^. First, we employed GSW multiyear surface water occurrence dataset with a pixel value above the 25% (represents seasonal water) and 75% (represents permanent water) threshold for selecting water observations. Then, we clipped the GSW water surface dataset by lake MWE masks in this study to achieve the permanent area (minimum) and seasonal area (maximum) of study lakes from 1984 to 2020.

#### Supply coefficient of lakes

The supply coefficient (sc) of a lake is the ratio of lake basin area to lake area (Eq. 1). The greater the supply coefficient of the lake is, the more the lake is affected by the river water regime in the recharge area and the greater change in lake water level and size.

1 $$sc=\frac{Are{a}_{basin}}{Are{a}_{lake}}$$

#### Population trend analysis

Further, we analyzed the population trend using the linear regression method^36^. We assume that the population of the Chinese lake basin varies linearly^37^. So, we used a linear slope to represent the population trend by the following equation.

2 $$k=\frac{{\sum }_{i=1}^{n}\left({t}_{i}-\bar{t}\right)\left({y}_{i}-\bar{y}\right)}{{\sum }_{i=1}^{n}{\left({t}_{i}-\bar{t}\right)}^{2}}$$

where *k* is the linear slope of the population trend of Chinese lake basins. When *k*>0, it indicates that the population is increasing, and vice versa. *t*_*i*_ is the given year corresponding to the population and *y*_*i*_ is the given population of year i. $\bar{t}$ and $\bar{y}$ represent the average value of year and population, respectively.

#### Drought index

The standardized precipitation evapotranspiration index (SPEI) based on precipitation and temperature data was used to extend the drought attribute of climate dataset in CODCLAB. SPEI can indicate the drought trend and has been widely used in the drought assessment and water resource management fields^38^. The applicability of SPEI to indicate drought monitoring has been proved in China^39^. In this study, a 3-month scale (equal to the time span of one season) of SPEI in the last 40 years (1980–2019) was computed to represent the seasonal drought severity of lake basins in CODCLAB.

## Data Records

The CODCLAB dataset is a reprocessing data set from publicly available data sources based on spatial analysis and mathematical statistics methods. All the publicly available data sources with physical and human-dimensional attributes are filtered through quality control. The principle of public data screening mainly considers data sets with ground validation and has close attention to natural sciences and humanities research. The CODCLAB dataset^40^ is available in three data formats, including tiff raster layers (Level 1), shapefiles (Level 2), and attribute tables (Level 3). The Level 1 data in tiff format stores the original static or time series rater dataset of CODCLAB, e.g., topography, climate, anthropogenic, and soils data set. Lake-basin scale characteristics assigned to the basins are stored in shapefiles associated with lake-basin polygons, such as supply coefficient of lakes, etc. Table 2 describes the naming rules for variables and units of the attribute value in separate shapefiles. All lake-basin attributes are provided in Level 3 tables associated with the lake ID, i.e., ‘Anth_CODCLAB.xlsx’ file, which stores anthropogenic information including lake ID, population density, GDP, etc. In addition to the above-mentioned CODCLAB_Level 1, Level 2, and Level 3, we also provide the CODCLAB of sub-basins for five large lakes and basic geographic information data in vector format, which are named CODCLAB_sub-basins^41^ and CODCLAB_Level 0^41^, respectively. The detailed data description of CODCLAB for different levels is shown in Table S2.

**Table 2:** Definitions of attributes in the CODCLAB.

Category	Attributes	Columns	Unit of values	Count
Hydrology	Lake extent	LWA + occurrence	km^2^	2
	Lake volume	Lake_vol	km^3^	1
	Residence time	Res_time	days	1
	Watershed area	Basin_area	km^2^	1
	Supply coefficient	SC	dimensionless	1
Topography	Elevation	Elevation	m	1
	Terrain slope	Slope	degrees	1
	Relief amplitude	RA	m	1
Climate	Temperature	Tem + year	0.1 °C	36
	Precipitation	Pre + year	0.1 mm	36
	Evapotranspiration	Eva + year	mm	36
	Pressure	Pres + year	pa	40
	Specific humidity	Shum + year	kg/kg	40
	Wind speed	Wind + year	m/s	40
	2m-air temperature	Temp + year	k	40
	Precipitation rate	Prec + year	mm/hr	40
	Seasonal SPEI	SPEI + season	dimensionless	4
Anthropogenic	Population count	Tpop + year	number	6
	Population density	Pd + year	number/km^2^	21
	Population trend	Pt	count/km^2^/5 yrs	1
	Nighttime lights	NTL + year	w/cm^2^/sr	21
	Human footprint	FP + year	dimensionless	2
	Gross domestic product	GDP + year	10^4^Yuan/km^2^	5
	Land cover/use	Type (cp *et al*.) + year	km^2^	279
Soils	Soil property	Attri (×12) + depth (×6)	Attri unit + cm	77
	Soil moisture	SM + year	m³/m³	16

### Hydrology dataset

**Fig. 4:**
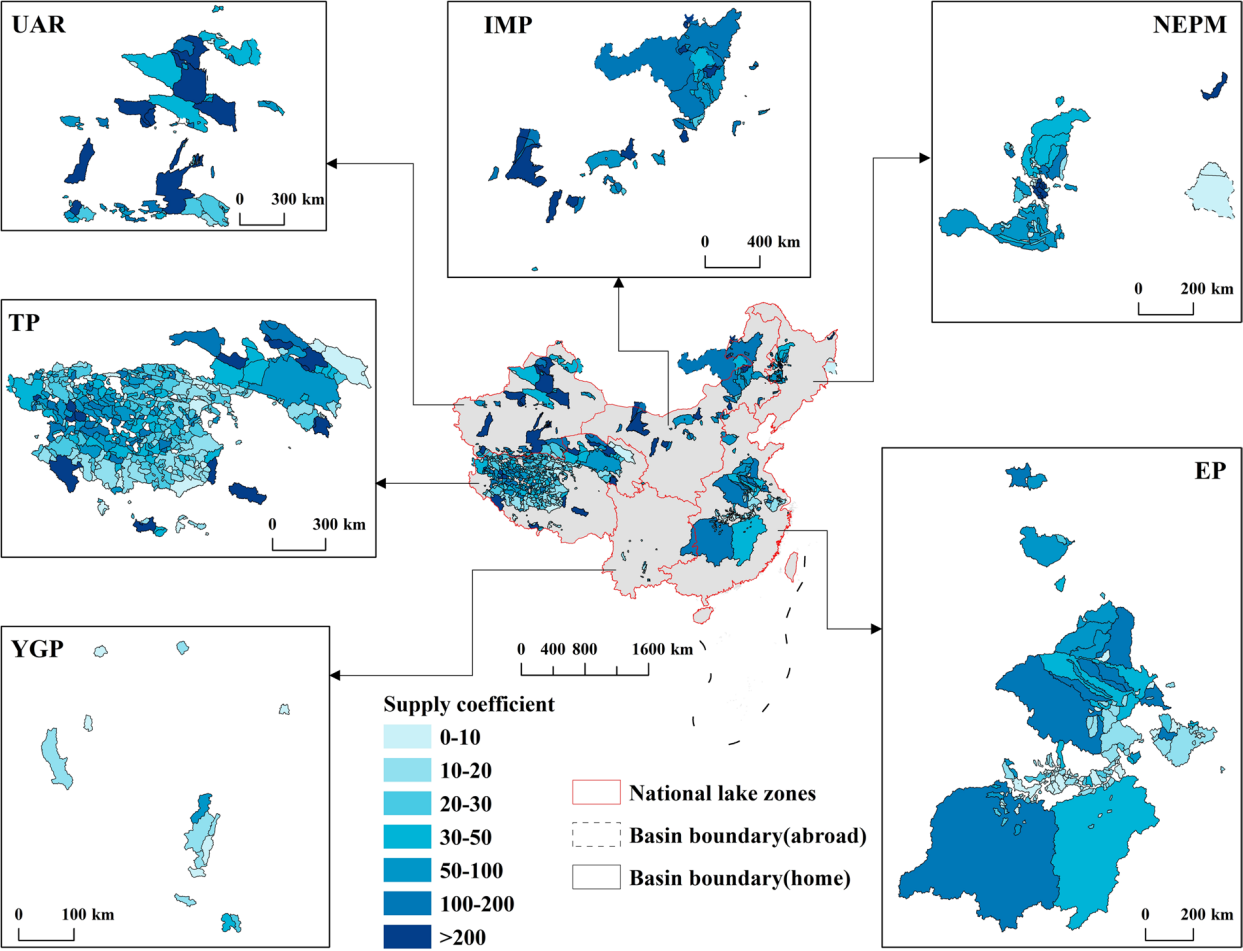
Supply coefficient of lakes based on max water extent displayed by six national lake zones.

The hydrology dataset of CODCLAB is the static vector data that reflects characteristics of lake basins at the stationary time scale, i.e., lake area, lake volume, residence time, etc. Usually, lake ID corresponds to the static variable in a one-to-one way, so we store this type of data in vector shapefiles combined with lake-basin polygons in the study. The supply coefficient of lakes obtained through calculation is shown as sample data records (Fig. 4). The supply coefficient of lakes showed significant spatial heterogeneity. Located in arid northwest China, the supply coefficient of lakes in the UAR zone was relatively high. However, the lakes in the humid areas of southwest and southeast China had a lower supply coefficient, i.e., the lakes in the YGP and EP lake zones (details in Table S1 and Fig. S1). The higher ratio of lake basin area to lake area (supply coefficient) in arid regions means that lakes in that region need more flowing water to recharge and sustain the lake water balances. In contrast, lakes in humid areas need fewer supplements. In addition, the range value of supply coefficient of lakes was calculated based on the permanent and seasonal lake area derived from water occurrence layer of GWS dataset (Figs. S2–S3).

### Topography dataset

Topography information of Chinese lake basins comprising elevation, slope, and relief amplitude is extremely useful for the hydrologic study of lakes or lake basins. In the CODCLAB dataset, all topography datasets are available in a three-level data organization with separate files (tiff raster, shapefile, and table format). For example, ‘Elevation_IDxx.tif’ file represents the Level 1 raster format dataset of elevation for the lake basin with IDxx. ‘Topo_CODCLAB.shp’ and ‘Topo_CODCLAB.xlsx’ store all the topography attributes of study lake basins in Level 2 and Level 3 data format, respectively.

### Climate dataset

**Fig. 5:**
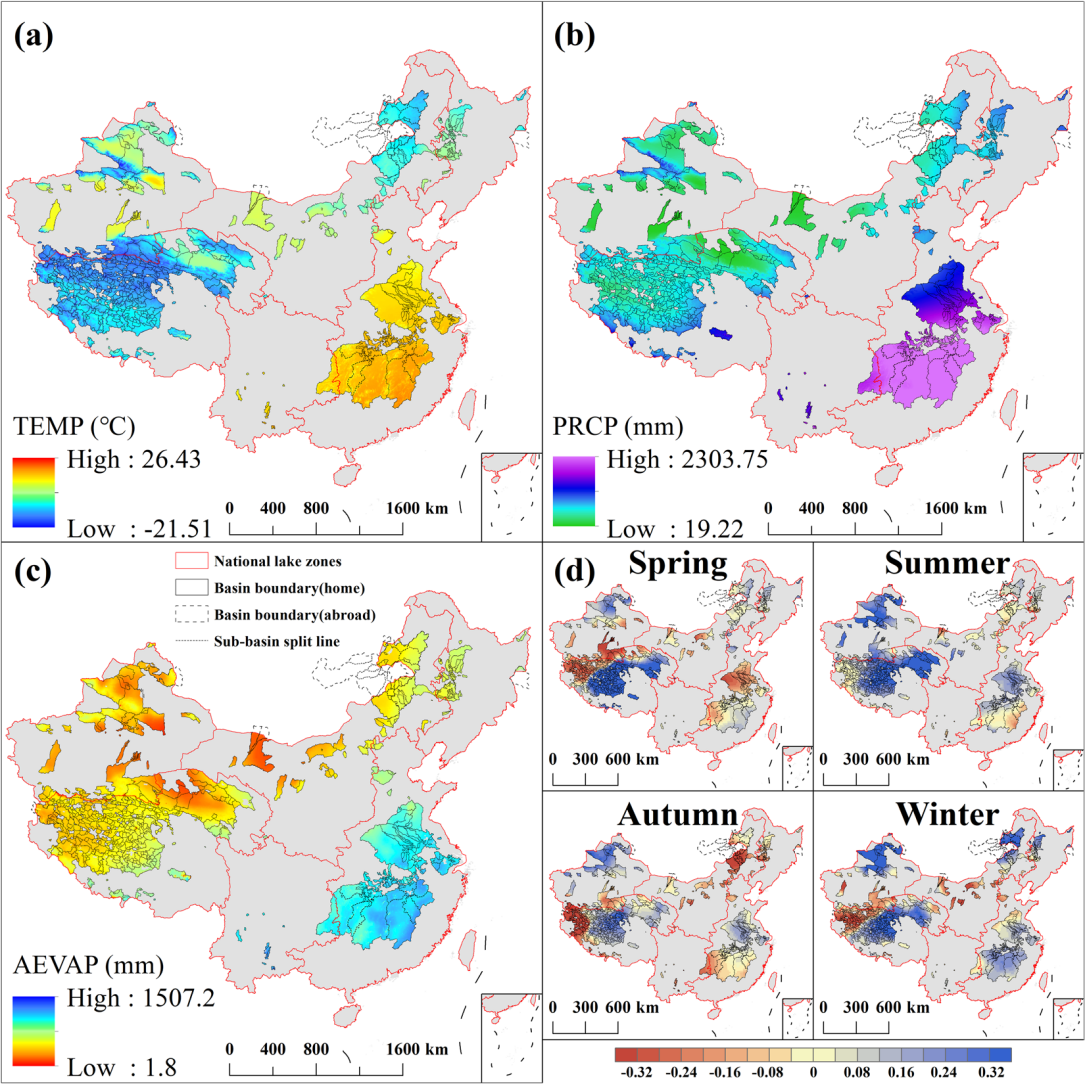
Climate data set of CLBCD. (a) Annual average temperature, (**b**) Annual precipitation, (**c**) Annual average actual evapotranspiration, and (**d**) Seasonal SPEI during the latest 40 years based on monthly temperature and precipitation.

The climate characteristics of CODCLAB show obvious spatial heterogeneity (Fig. 5). The mean annual temperature for China’s lake basins ranged from −21.51 to 26.43 °C, with an average of 7.51 °C. The lowest value corresponds to the location of lake basins in the TP zone, and the highest value was observed at a location of lake basins in the UAR zone (Fig. 5a). The mean annual total precipitation ranged from 19.22 to 2303.75 mm, with an average value of 679.01 mm, and the minimum and maximum values corresponded to locations in the lake-basins in TP and southeast part of the lake basins in EP (Poyang Lake basin and Dongting Lake basin), respectively (Fig. 5b). The mean annual actual evapotranspiration (AEVAP) ranged from 1.8 to 1507.2 mm, with an average of 427.59 mm (Fig. 5c), and the distribution of AEVAP of CODCLAB is positively correlated with precipitation and temperature (Fig. 5). The drought trend of China lake-basins on seasonal scales is illustrated in Fig. 5d. It reflects temporal and spatial characteristics of seasonal drought on a time scale of 3 months. As a result, the lake basins tend to get drier in the northwestern part of TP and the central and western part of IMP during spring, autumn, and winter. The lake basins in EP also show a significant drying trend in the spring and fall. In contrast, the lake basins of western TP, northern UAR, and western NEMP became significantly wet. Interestingly, lake basins with a perennially dry tendency tend to have lower average temperatures and less precipitation and evaporation (e.g., Western IMP, Southwest UAR, and Northwest TP).

### Anthropogenic dataset

**Fig. 6:**
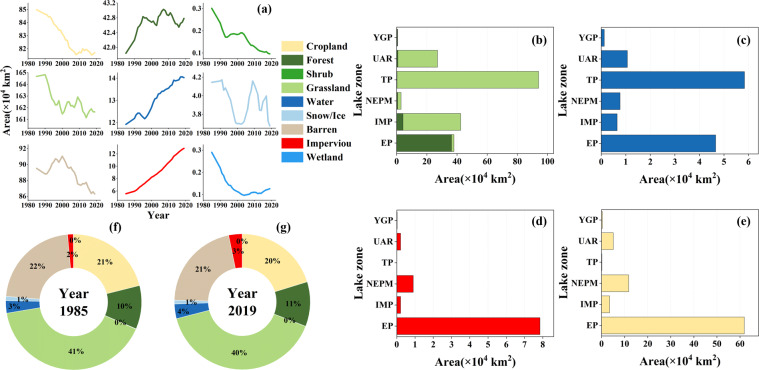
LUCC of CODCLAB during 1985–2019. (**a**) Dynamics of LUCC by nine types. (**b**,**c**,**d**,**e**) Average area of green land, water, urban, and cropland in national lake zones from 1985–2019, which represent land use/cover patterns driven by nature and humans, respectively. (**f**,**g**) Composition of nine land use/cover types in 1985 and 2019.

**Fig. 7:**
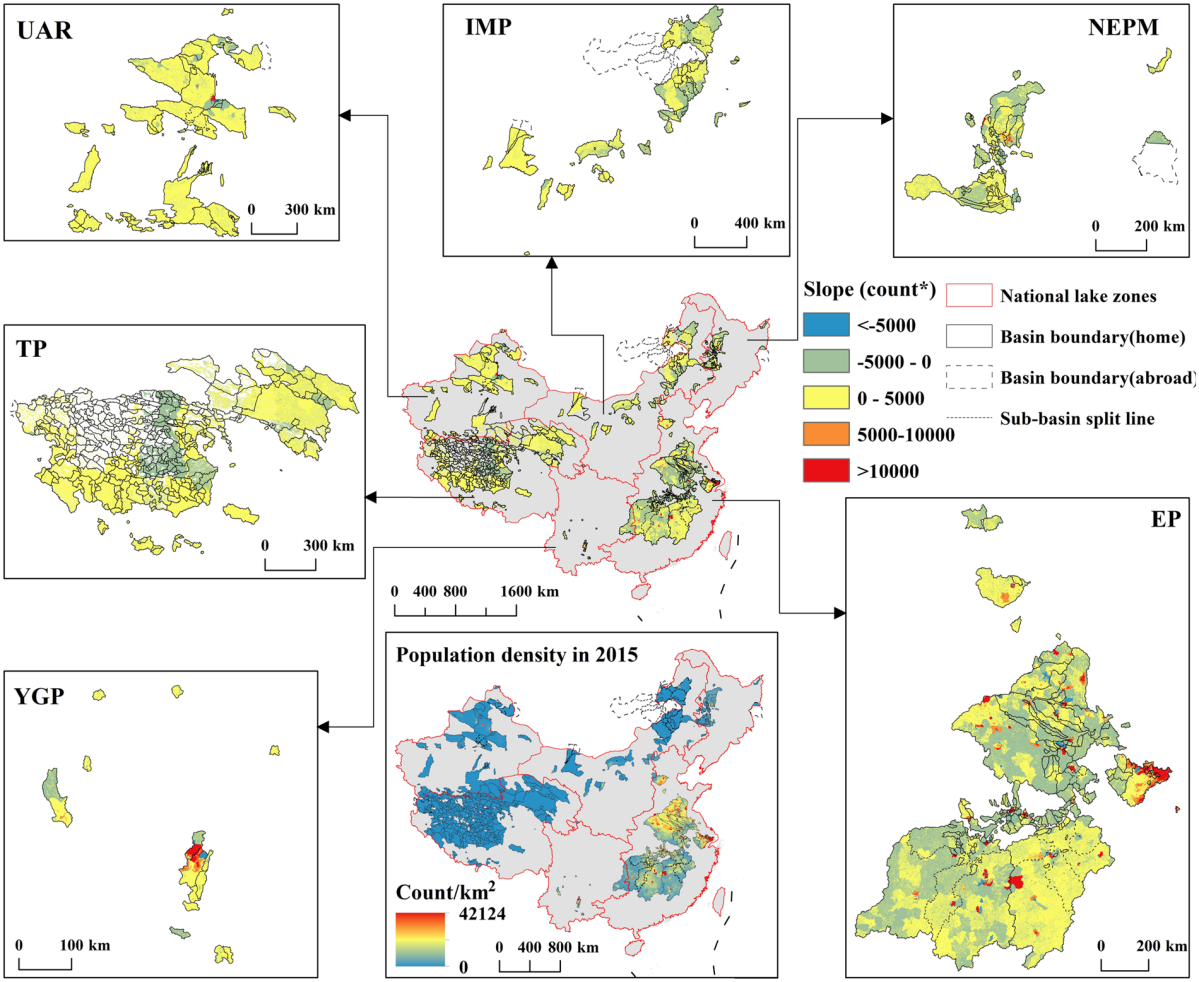
Population density in 2015 and linear slope of population trends displayed by six national lake zones during 1990–2015. *count represents the population count of the linear slope unit. And by the significance test, the t-test result p-value is 0.03.

Human activity can substantially alter anthropogenic pressures on lake hydrology and eco-environment. We take land use/cover and population density as examples to state the time series anthropogenic data records of CODCLAB stored in the format of a tiff raster (Figs. 6–7). Land use/cover change (LUCC) of lake basins gives the watershed perspective to understand the impacts of anthropogenic pressures on lake hydrology. Green land, such as forests and grasslands, accounts for half of China’s natural lacustrine basins (Fig. 6f,g). On the other hand, urban impervious surface and cropland dominated by human activities account for 23% of China’s lacustrine basins (Fig. 6f,g). In the past 35 years, forest, water bodies, and urban land use/cover have increased continuously, while the other six land types have fluctuated and declined (Fig. 6a). The intensity of human activities also shows obvious spatial heterogeneity in different lake zones (Fig. 6). Urban impervious surface and cropland dominate the lake basins in the eastern plain of China (Fig. 6d,e). While water and grassland almost occupy the whole composition of the lake basin area in the Tibetan Plateau (Fig. 6b,c).

The spatial distribution of population density between eastern and western lake basins is highly consistent with the land use/cover difference (Fig. 7). The high population density distribution in the EP lake zone resulted in strong human intervention (i.e., urban land and cropland change) in the lake basins. Further, the lake basins with the fastest population growth are the Taihu and Dianchi lake-basin with over 10000 count/km^2^/5 yrs (Fig. 7). In addition, some low population density basin areas in the six national lake zones are losing population. In summary, the population change rate in the lake basins of China is proportional to the population density.

### Soils dataset

Soils dataset of CODCLAB includes three-dimensional soil texture information and soil moisture. The soil dataset can be applied in many research fields, including agriculture, hydrology, climate, ecology, and environment. CODCLAB offers sand, silt, clay contents, etc., in each lake basin and at multiple depths of 0–5, 5–15, 15–30, 30–60, 60–100, and 100–200 cm. All soil data sets are available in a three-level data organization with separate files (tiff raster, shapefile, and table format). In addition, CODCLAB applies ‘attributes + depth’ to assign soil information to each lake basin.

## Technical Validation

Major CODCLAB variables reformat existing source data into the geospatial frameworks of the lake basin of China apart from a few reanalysis data. The quality of original datasets (known as source data) is already validated by other independent studies as follows table (Table 3). Furthermore, we still present the following local validation of global dataset and cross validation of localized dataset in China to illustrate the accuracy of CODCLAB.

**Table 3:** Overall accuracy of source data and applicability evaluation in China.

Name	Variables	Overall accuracy assessment description	Reference	Accuracy evaluation description in China	Reference
JRC GSW	Lake extent	Permanent water with 99.6% (TM), 99.5% (ETM+) and 99.7% (OLI), respectively. Seasonal water with 98.8% (TM), 98.4% (ETM+) and 98.5% (OLI), respectively	Pekel, *et al*.^24^	During cross-validation with JRC GSW, the average producer’s accuracy and user’s accuracy of water are 0.933 and 0.998, respectively	Tang, *et al*.^50^
SRTM1 DEM	Elevation	Global statistics for a mean difference of 3 m and a standard deviation of 16 m	Berry, *et al*.^51^	7.6–25 m over five different geographical localities in China Vertical mean difference (0.60 m) and RMSE (2.78 m) using GPS as a reference in northeastern China Sichuan (ME = 2.95, RMSE = 10.04 m), Xinjiang A (ME = 2.87 m, RMSE = 3.29 m), Xinjiang B (ME = 1.85 m, RMSE = 6.11 m), Inner Mongolia (ME = 1.05 m, RMSE = 3.16 m)	Li, *et al*.^52^ Dong, *et al*.^53^ Han, *et al*.^54^
CMFD*	Climate	—		CMFD has close-to-zero mean bias error (MBE), lower root mean square error (RMSE), and higher R^2^ than GLDAS for almost all variables	He, *et al*.^43^
WorldPop Collection (Mainland China)*	Population density	—		Median absolute deviation (MAD) of population density (mean of squared residuals) for each year is 1.64, 1.64, and 2.32 for 1990, 2000, and 2010	Gaughan, *et al*.^55^
NTL	Nighttime light	R-squared (R^2^): pixel level, 0.87; city level, 0.95	Chen, *et al*.^45^	R^2^ = 0.72, RMSE = 2.15, at pixel level	Chen, *et al*.^45^
CLCD*	Landuse	—		Overall accuracy 79.31%	Yang and Huang^47^
SMC_V3*	Soil moisture	—		Bias: 0.057 m^3^/m^3^, unbiased RMSE: 0.056, correlation coefficient (R): 0.84	Meng, *et al*.^49^

*The source data is the localization variable dataset in China.

### Local validation

Most of the source data of CODCLAB are localized in China. A small amount of global data used by CODCLAB has been widely applied in China, and some local validation accuracy has been found to support the CODCLAB (e.g., GSW, SRTM, and NTL shown in Table 3).

1. Validation of lake extent derived from the GSW dataset
  We randomly selected six lakes from different national lake zones as validation examples (Fig. 8). We validated their lake area extraction results by comparing GSW retrieve results and manual digitizing results through high-resolution remote sensing images of Sentinel-2 satellite with different periods. The validation result was shown in Fig. 8 combined with total R-squared (R^2^) and mean absolute percent error (MAPE; Eq. (3)) of 0.99 and 2.56%, respectively.
  3 $$MAPE=\frac{1}{n}{\sum }_{i=1}^{n}\left|\frac{S{1}_{i}-S{2}_{i}}{S{2}_{i}}\right|\times 100 \% $$
  where S1 is the lake area obtained digitally from Sentinel-2 images and S2 is the lake area derived from GSW retrievals. And i is the selected date of validation, and n denotes the number of selected dates for the one lake to validate.

**Fig. 8:**
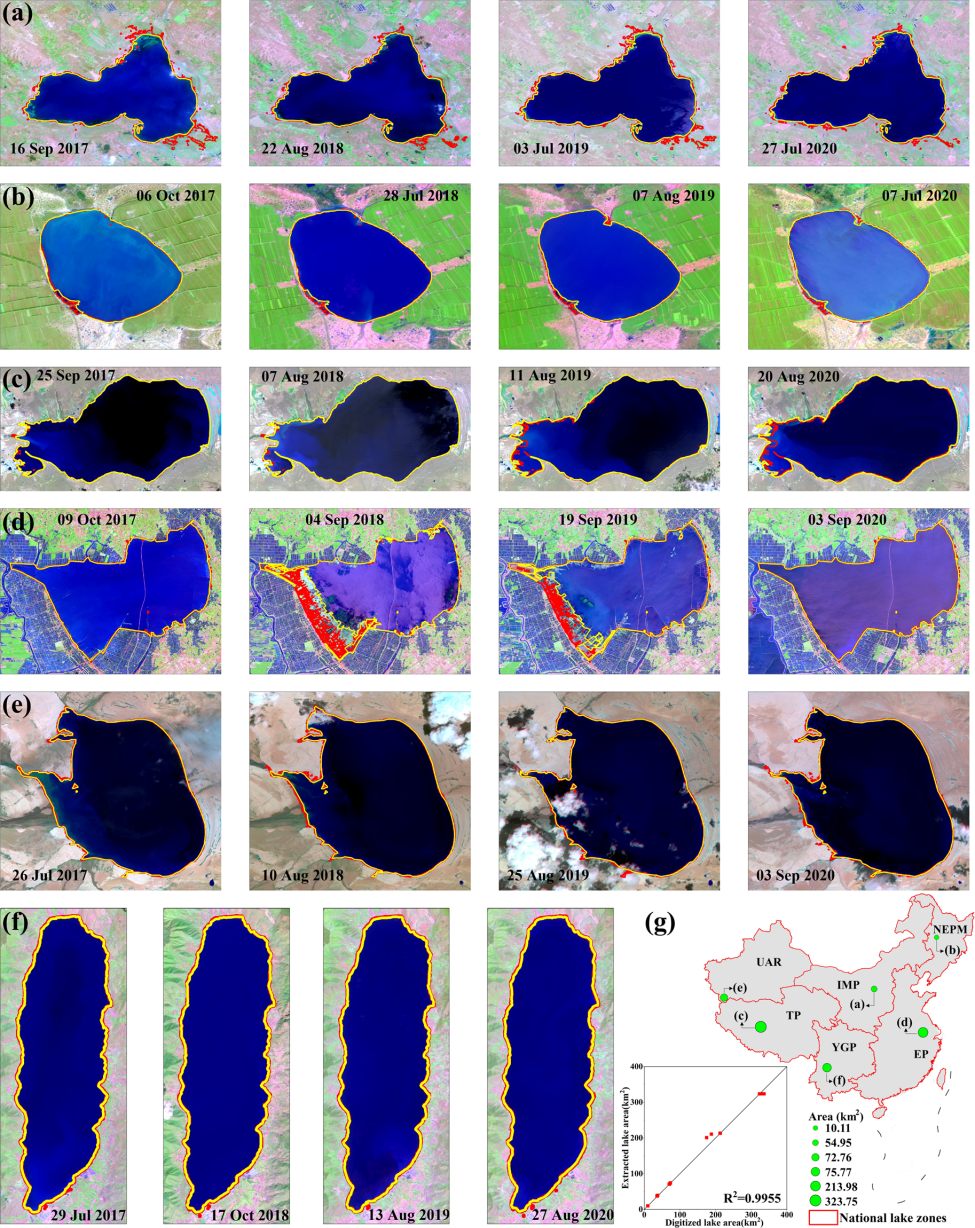
Validation of lake surface area extraction based on high-resolution satellite images. The yellow line is the water extent from Sentinel-2, and the red line is the water extent from GSW. Six sample lakes include (**a**) Hongjiannnao in IMP, (**b**) Jiangshenpao in NEPM, (**c**) DagzeCo in TP (**d**) Shijiu lake in EP, (**e**) Kule lake in UAR, and (**f**) Chenghai in YGP. (**g**) The location of six sample lakes in different national lake zones and same-day pairwise comparisons between lake area extraction.
2. Validation of elevation derived from the SRTM1 DEM dataset
  Previous studies have validated the accuracy of SRTM at regional scales in China (Table 3). Further, we utilized the Ice, Cloud, and land Elevation Satellite (ICESat) footprints to validate the SRTM data in our CODCLAB dataset at the lake basin scale. The spatial distribution of the ICESat footprints shows that the validate points can cover all lake zones and almost all lake basins (Fig. 9a). The scatter plot of verification points compares the consistent distribution of the SRTM1 DEM data and ICESat elevation data (Fig. 9b). The results show that the elevation of CODCLAB derived from SRTM1 DEM dataset has a better performance with an R^2^ of 0.99 and an RMSE of 8.07 m. In addition, the SRTM1 DEM data have a positive 1:1 relationship with the ICESat elevation data according to most verification points around the non-bias (1:1) line (Fig. 9).

**Fig. 9:**
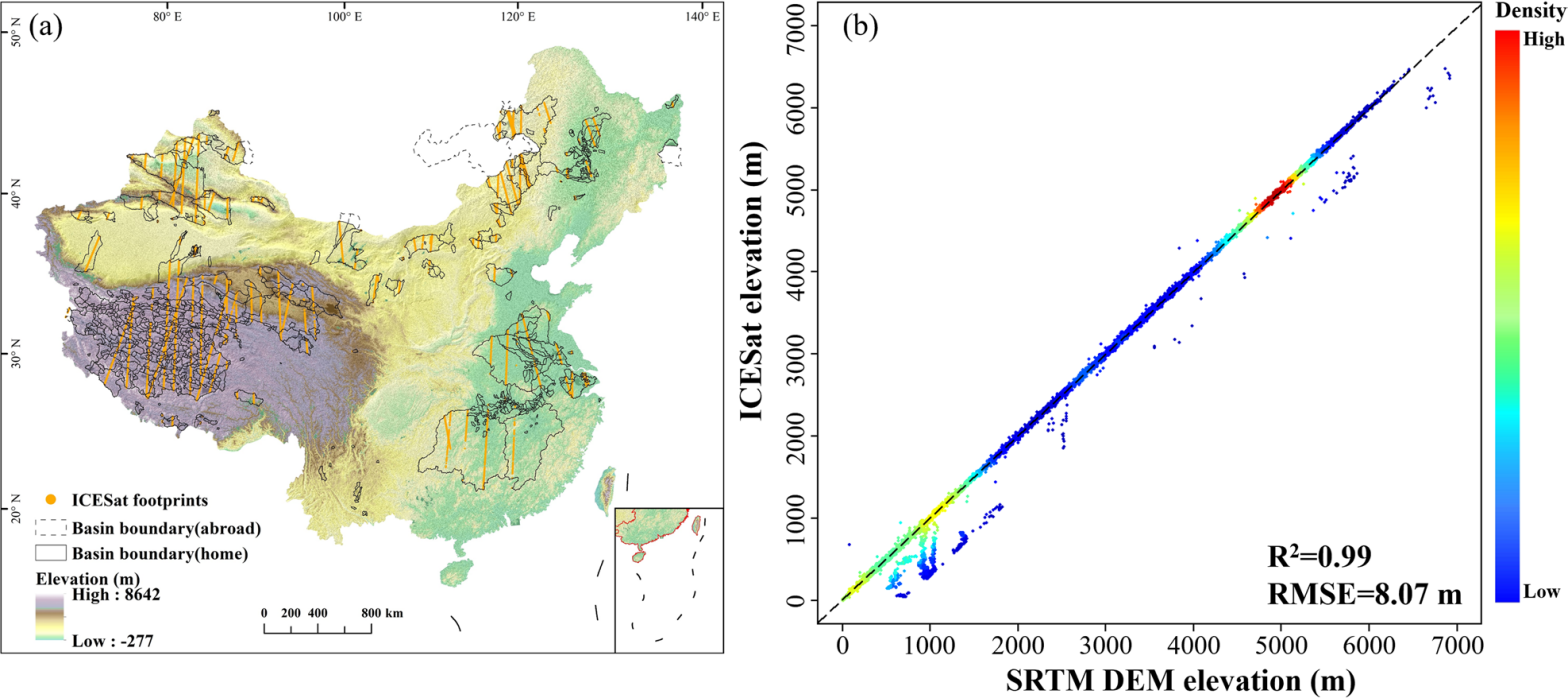
(**a**) Spatial distribution of the ICESat footprints. (**b**) The comparison between the SRTM DEM elevation and ICESat elevation in 2003. The dashed line denotes the 1:1 line, and the points are colored by their respective density, which red to blue indicate density from high to low.
3. Validation of nighttime lights derived from the global NTL dataset

**Fig. 10:**
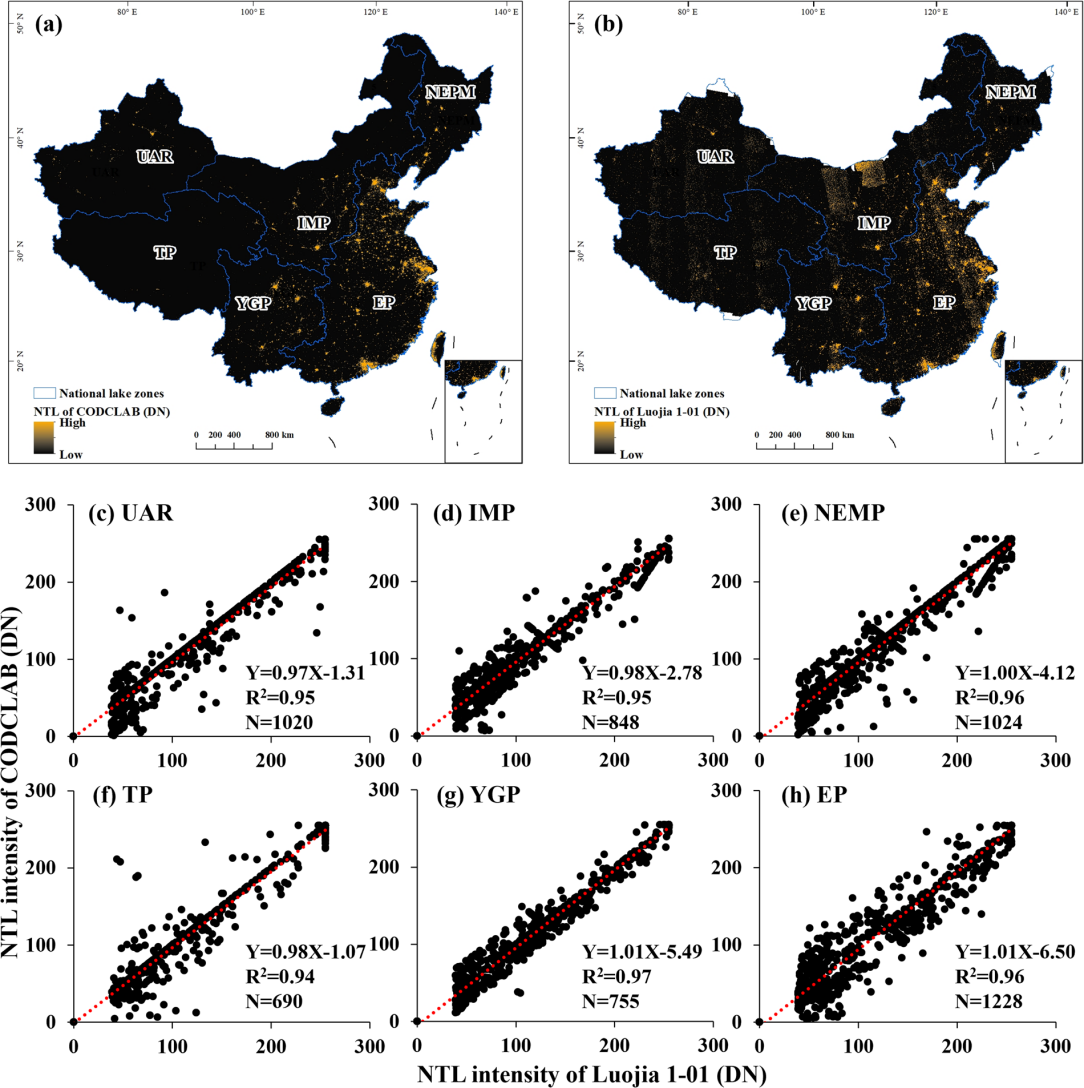
Comparison of NTL intensity from (**a**) CODCLAB and (**b**) Luojia 1-01. Local validation of NTL from selected sites of national lake zones over (**c**) UAR, (**d**) IMP, (**e**) NEMP, (**f**) TP, (**g**) YGP, and (**h**) EP in 2018. The red dash line denotes the linear fitting curve, and N is the number of sample points.

In this study, the Luojia 1-01 nighttime light imagery developed by Wuhan University (http://59.175.109.173:8888/) was employed to verify the accuracy of the global NTL dataset in China. The Luojia 1-01 has a fine spatial resolution compared to the NTL dataset of CODCLAB composited by DMSP-OLS and NPP-VIIRS data. The Luojia 1-01 launched in 2018 also localized in China, and it is well suited for validating global NTL data. As shown in Fig. 10, the NTL of CODCLAB and the NTL derived from Luojia 1-01 have a consistent spatial pattern at both national and regional scales. Among the national validation points within six lake zones (Fig. 10c–h), we find that the accuracy of NTL of CODCLAB in these lake zones is acceptable, and no significant variation. YGP has the highest accuracy with an R^2^ of 0.97, followed by NEMP and EP (R^2^ = 0.96). The rest of the lake zones all have an accuracy higher than 0.93, which means the NTL intensity of CODCLAB is similar to the Luojia 1-01 at the pixel level.

### Cross validation

**Fig. 11:**
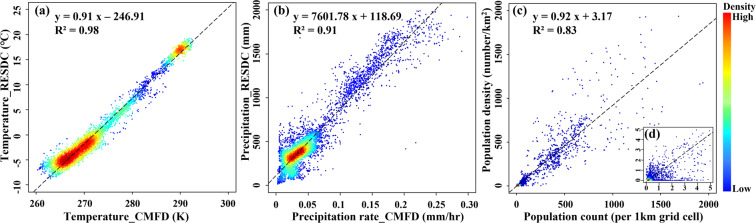
Cross-validation of datasets in CODCLAB. (**a**) REDSC derived Celsius temperature versus CMFD derived Kelvin temperature. (**b**) REDSC derived total precipitation versus CMFD derived precipitation rate. (**c**) RESDC derived population count versus WorldPop derived population density. (**d**) Zoom insert map presents the comparisons range from 0 to 5. The dashed line is plotted as the fitting relationship. The points are colored by their respective density, and red to blue indicate density from high to low.

We selected three groups of variables with multiple data sources for cross validation of CODCLAB (Fig. 11). The R^2^ values of the three groups of variables are all greater than 0.8, which means that each group of variables has a strong correlation. The temperature of all study lake basins derived from the RESDC and CMFD has the highest relevancy (R^2^ = 0.98). For precipitation, there is no same variable from multiple sources, yet the precipitation of REDSC still has a strong correlation with the precipitation rate of CMFD (R^2^ = 0.91). Similarly, population density and population count per square kilometer of different data sources also have a strong correlation (R^2^ = 0.83). Therefore, the original validated datasets in independent research can be conducted to manifest the consistency and reliability of CODCLAB due to the cross validation.

## Usage Notes

The CODCLAB can be used in a suite of research areas relating to hydro-environmental studies at the lake basin scale of China. For example, the climate parameters provided by the CODCLAB can be used to analyze the effects of basin-scale climate change on the hydrological dynamics of lakes. Second, the anthropogenic attributes of CODCLAB can be applied to understand the impact of human activities on lake basins. In addition to employing the variables of different types individually, CODCLAB can also be applied by combining multiple variables in comprehensive studies. For instance, we need to invoke both anthropogenic and hydrological variables of CODCLAB to understand the impact of population change on lake dynamics.

The data files are formatted as tiff raster layers (CODCLAB_Level 1), shapefiles (CODCLAB_ Level 2), and attribute tables (CODCLAB_Level 3) based on the three-level organization. It still requires users to decide which level of data and which type of variables to employ. In addition to the uniform resolution dataset (CODCLAB_1km)^41^, users also need to consider the differences in temporal and spatial resolution between different CODCLAB variables.

As the potential for future application, the CODCLAB can be used to increase research efficiency by allowing users to quickly achieve multi-source data with the common georeference for location-specific studies. Suppose that future data users can describe lake or basin changes with co-located hydrometeorological and anthropogenic data based on one-stop resources served by CODCLAB.

## Supplementary information

Supplementary Information of CODCLAB

## Data Availability

Two core tools applied in the study were ‘Spatial join’ and ‘Zonal Statistics’ provided by ESRI’s. ArcGIS 10.7 software package. In addition, the customized batch steps of reprocessing data, including lake area extraction and raster attribute extraction, were programmed using Python 2.7 scripts which were provided in our data set named ‘Lake_area_extraction.py’ and ‘Raster_attribute_ extraction.py’, respectively^41^.

## References

[1] Fergus CE (2021). National framework for ranking lakes by potential for anthropogenic hydro-alteration. Ecological Indicators.

[2] O’Beirne M (2017). Anthropogenic climate change has altered primary productivity in Lake Superior. Nature communications.

[3] Perga M-E (2015). High-resolution paleolimnology opens new management perspectives for lakes adaptation to climate warming. Frontiers in Ecology and Evolution.

[4] Peter KH, Nnko HJ, Mubako S (2020). Impacts of anthropogenic and climate variation on spatiotemporal pattern of water resources: A case study of Lake Babati, Tanzania. Sustainable Water Resources Management.

[5] Pokhrel Y (2012). Incorporating anthropogenic water regulation modules into a land surface model. Journal of Hydrometeorology.

[6] Zhang G (2019). Regional differences of lake evolution across China during 1960s–2015 and its natural and anthropogenic causes. Remote Sensing of Environment.

[7] USEPA. National lakes assessment 2012: a collaborative survey of lakes in the United States. 2016.

[8] Mao D, Cherkauer KA (2009). Impacts of land-use change on hydrologic responses in the Great Lakes region. Journal of Hydrology.

[9] Shirmohammadi B (2020). Scenario analysis for integrated water resources management under future land use change in the Urmia Lake region, Iran. Land Use Policy.

[10] Schindler D (2009). Lakes as sentinels and integrators for the effects of climate change on watersheds, airsheds, and landscapes. Limnology and Oceanography.

[11] Sayer CA, Carr JA, Darwall WR (2019). A critical sites network for freshwater biodiversity in the Lake Victoria Basin. Fisheries Management and Ecology.

[12] Singh P, Kumar A, Mishra S (2021). Performance evaluation of conservation plan for freshwater lakes in India through a scoring methodology. Environment, Development and Sustainability.

[13] Linke S, Hermoso V, Januchowski‐Hartley S (2019). Toward process‐based conservation prioritizations for freshwater ecosystems. Aquatic Conservation: Marine and Freshwater Ecosystems.

[14] Meyer MF, Labou SG, Cramer AN, Brousil MR, Luff BT (2020). The global lake area, climate, and population dataset. Scientific data.

[15] Messager ML, Lehner B, Grill G, Nedeva I, Schmitt O (2016). Estimating the volume and age of water stored in global lakes using a geo-statistical approach. Nature communications.

[16] Linke S (2019). Global hydro-environmental sub-basin and river reach characteristics at high spatial resolution. Scientific data.

[17] Cai X, Feng L, Hou X, Chen X (2016). Remote sensing of the water storage dynamics of large lakes and reservoirs in the Yangtze River Basin from 2000 to 2014. Scientific reports.

[18] Feng L (2012). Assessment of inundation changes of Poyang Lake using MODIS observations between 2000 and 2010. Remote Sensing of Environment.

[19] Lei Y (2014). Response of inland lake dynamics over the Tibetan Plateau to climate change. Climatic Change.

[20] Song C, Huang B, Ke L (2013). Modeling and analysis of lake water storage changes on the Tibetan Plateau using multi-mission satellite data. Remote Sensing of Environment.

[21] Chen T (2022). Remote sensing estimation of the flood storage capacity of basin-scale lakes and reservoirs at high spatial and temporal resolutions. Science of The Total Environment.

[22] Wen Z (2020). A national-scale data set for dissolved carbon and its spatial pattern in lakes and reservoirs across China. Scientific data.

[23] Liu F (2020). High-resolution and three-dimensional mapping of soil texture of China. Geoderma.

[24] Pekel J-F, Cottam A, Gorelick N, Belward AS (2016). High-resolution mapping of global surface water and its long-term changes. Nature.

[25] Chen T (2021). Estimating seasonal water budgets in global lakes by using multi-source remote sensing measurements. Journal of Hydrology.

[26] Busker T (2019). A global lake and reservoir volume analysis using a surface water dataset and satellite altimetry. Hydrology and Earth System Sciences.

[27] Lu S (2019). Time series of the Inland Surface Water Dataset in China (ISWDC) for 2000–2016 derived from MODIS archives. Earth System Science Data.

[28] Zhu J, Song C, Wang J, Ke L (2020). China’s inland water dynamics: The significance of water body types. Proceedings of the National Academy of Sciences.

[29] Zhang, W. & Song, C. The Spatial Distribution and Dynamics of Lakes in China: Progress in Remote Sensing Monitoring at National Scale and New Inventory of the Maximum Lake Extent and Change Trajectory. *National Remote Sensing Bulletin*, 1-14, 10.11834/jrs.20211290 (2021).

[30] Hammer, U. T. *Saline lake ecosystems of the world*. Vol. 59 (Springer Science & Business Media, 1986).

[31] Ma, R. *et al*. A half‐century of changes in China’s lakes: Global warming or human influence? **37** (2010).

[32] Lehner B, Grill G (2013). Global river hydrography and network routing: baseline data and new approaches to study the world’s large river systems. Hydrological Processes.

[33] Lehner, B. HydroBASINS: Global watershed boundaries and sub-basin delineations derived from HydroSHEDS data at 15 second resolution — Technical documentation version 1.c, https://hydrosheds.org/page/hydrobasins (2014).

[34] Farr, T. G. *et al*. The shuttle radar topography mission. *Reviews of geophysics* **45** (2007).

[35] O’Callaghan, J. F., Mark, D. M. J. C. v., graphics, & processing, i. The extraction of drainage networks from digital elevation data. **28**, 323–344 (1984).

[36] Weisberg, S. *Applied linear regression*. Vol. 528 (John Wiley & Sons, 2005).

[37] Lo C, Welch R (1977). Chinese urban population estimates. Annals of the Association of American Geographers.

[38] Vicente-Serrano SM, Beguería S, López-Moreno JI (2010). A multiscalar drought index sensitive to global warming: the standardized precipitation evapotranspiration index. Journal of climate.

[39] Zhao H (2017). Timescale differences between SC-PDSI and SPEI for drought monitoring in China. Physics and Chemistry of the Earth, Parts A/B/C.

[40] Chen T (2022). figshare.

[41] Chen T (2022). figshare.

[42] Ma N, Szilagyi J, Zhang Y, Liu W (2019). Complementary‐relationship‐based modeling of terrestrial evapotranspiration across China during 1982–2012: Validations and spatiotemporal analyses. Journal of Geophysical Research: Atmospheres.

[43] He J (2020). The first high-resolution meteorological forcing dataset for land process studies over China. Scientific Data.

[44] Tatem AJ (2017). WorldPop, open data for spatial demography. Scientific data.

[45] Chen Z (2021). An extended time series (2000–2018) of global NPP-VIIRS-like nighttime light data from a cross-sensor calibration. Earth System Science Data.

[46] Venter O (2016). Global terrestrial Human Footprint maps for 1993 and 2009. Scientific data.

[47] Yang J, Huang X (2021). The 30 m annual land cover dataset and its dynamics in China from 1990 to 2019. Earth System Science Data.

[48] Liu, F. *et al*. Developing high resolution National Soil Information Grids of China. *Science Bulletin* (2021). 10.1016/j.scib.2021.10.013 36546081

[49] Meng X (2021). A fine-resolution soil moisture dataset for China in 2002–2018. Earth System Science Data.

[50] Tang, H. *et al*. Large-Scale Surface Water Mapping Based on Landsat and Sentinel-1 Images. **14**, 1454 (2022).

[51] Berry, P., Garlick, J. & Smith, R. J. R. S. O. E. Near-global validation of the SRTM DEM using satellite radar altimetry. **106**, 17–27 (2007).

[52] Li P (2013). Evaluation of ASTER GDEM using GPS benchmarks and SRTM in China. International Journal of Remote Sensing.

[53] Dong, Y., Chang, H.-C., Chen, W., Zhang, K. & Feng, R. J. G. I. Accuracy assessment of GDEM, SRTM, and DLR-SRTM in Northeastern China. **30**, 779–792 (2015).

[54] Han, H., Zeng, Q. & Jiao, J. J. R. S. Quality assessment of TanDEM-X DEMs, SRTM and ASTER GDEM on selected Chinese sites. **13**, 1304 (2021).

[55] Gaughan AE (2016). Spatiotemporal patterns of population in mainland China, 1990 to 2010. Scientific Data.

