# Supplementary material for: A comprehensive data set of physical and human-dimensional attributes for China’s lake basins
Source: Sci Data. 2022 Aug 25;9:519. doi: 10.1038/s41597-022-01649-z (PMC9411201; doi:10.1038/s41597-022-01649-z)
Supplement: Supplementary file 1 — Supplementary Information of CODCLAB [file 41597_2022_1649_MOESM1_ESM.docx]

**Supplementary Information**

**A comprehensive data set of physical and human-dimensional attributes for China’s lake basins**

Tan Chen^1^, Chunqiao Song^1^, Chenyu Fan^1,2^, Jian Cheng^3^, Xuejun Duan^1^, Lei Wang^1^, Kai Liu^1^, Shulin Deng^4^, Yue Che^5^

1. Key Laboratory of Watershed Geographic Sciences, Nanjing Institute of Geography and Limnology, Chinese Academy of Sciences, Nanjing 210008, China

2. College of Resources and Environment, University of Chinese Academy of Sciences, Beijing 100049, China

3. School of Geography and Ocean Science, Nanjing University, Nanjing 210023, China

4. School of Geography and Planning, Nanning Normal University, Nanning 530001, China

5. Shanghai Key Lab for Urban Ecological Processes and Eco-Restoration, School of Ecological and Environmental Sciences, East China Normal University, Shanghai 200241, China

**Contents of this file:**

Table S1-S2

Figure S1-S3


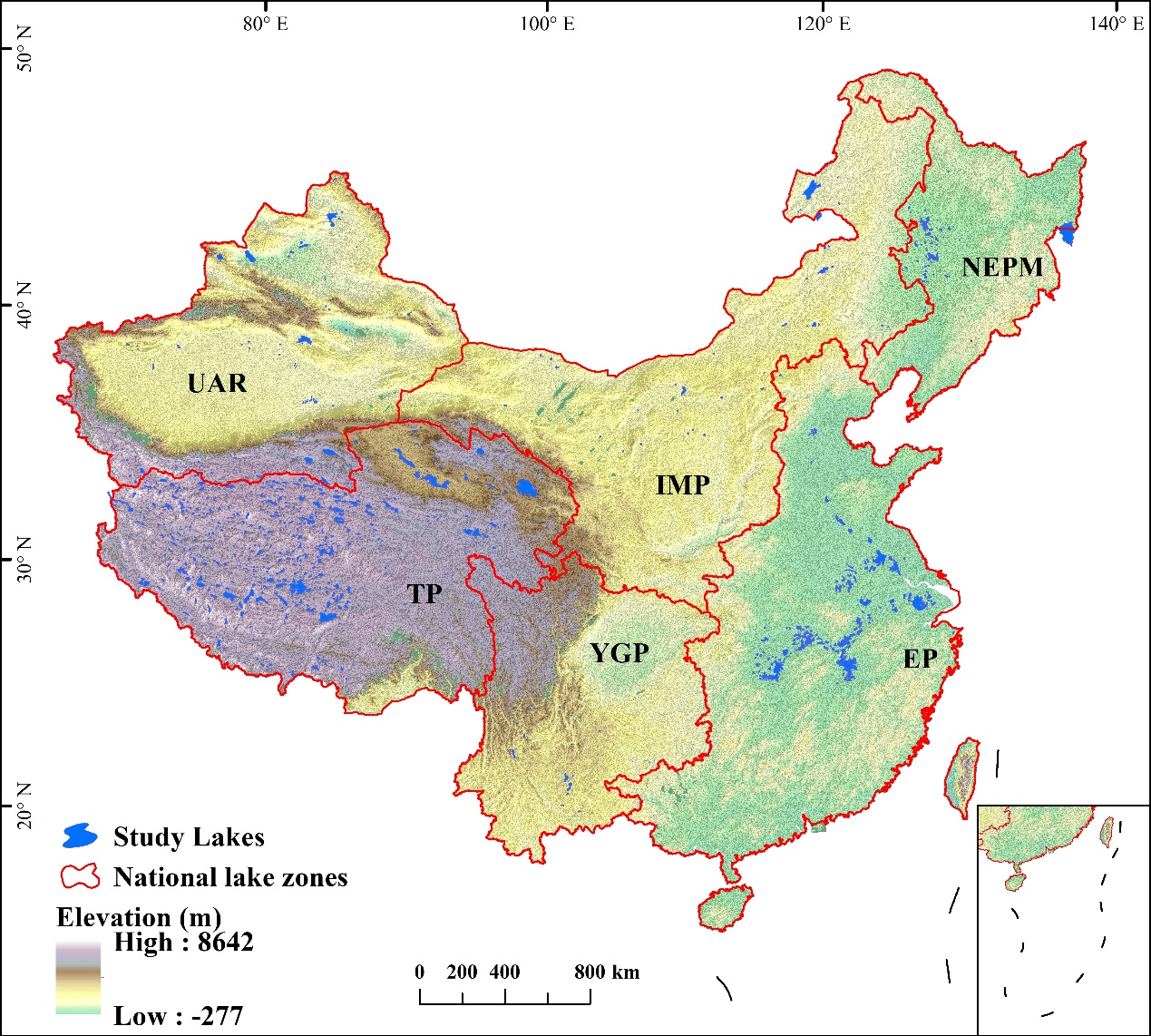


Fig. S1 The distribution of study lakes (> 10 km^2^) with elevation gradients based on SRTM in six lake zones. National lake zones include Yunnan-Guizhou Plateau (YGP), Tibetan Plateau (TP), Uygur Autonomous Region (UAR), Inner Mongolia Plateau (IMP), Northeast Plains and Mountains (NEPM), and Eastern Plains (EP) according to the geographical division of China with different altitude, climate, topography status.

Table S1 Summary of lakes above 10 km^2^ in different lake zones of China.

| Zones | Number | Total area (km^2^) | Mean area (km^2^) | Min area (km^2^) | Max area (km^2^) |
| --- | --- | --- | --- | --- | --- |
| TP | 411 | 46245.73 | 112.52 | 10.09 | 4440.61 |
| UAR | 77 | 8028.39 | 104.26 | 10.01 | 1116.82 |
| IMP | 48 | 5004.71 | 104.26 | 10.39 | 2305.44 |
| NEMP | 60 | 7248.11 | 120.80 | 10.04 | 4669.42 |
| EP | 157 | 21637.90 | 137.82 | 10.04 | 3616.34 |
| YGP | 14 | 1112.40 | 79.46 | 11.18 | 290.30 |
| Total | 767 | 89277.24 | 116.40 | 10.01 | 4669.42 |

Table S2 Data description of CODCLAB in different levels of organization

| **CODCLAB_Level 0**  Basic geographic information data in vector format. | |
| --- | --- |
| Name | Data description |
| Lake_basin_boundary | Lake basin boundaries with geographic reference |
| Lake_subbasin_boundary | Lake sub-basin boundaries with geographic reference |
| Lake_MAXextant | Maximum inundation area of lakes with geographic reference |
| National_lake_zones | Boundary of national lake zones with geographic reference |

| **CODCLAB_Level 1**  Original tiff raster layers accompanied with independent lake-basin ID. | | | | | | | |
| --- | --- | --- | --- | --- | --- | --- | --- |
| Category | | Attributes | | Data layers | | Number | |
| Topography | | Elevation | | Elevation | | 1 | |
|  |  | Terrain slope | | Slope | | 1 | |
|  |  | Relief amplitude | | RA | | 1 | |
| Climate | | Temperature | | Tem+year | | 36 | |
|  |  | Precipitation | | Pre+year | | 36 | |
|  |  | Evapotranspiration | | Eva+year | | 432 | |
|  |  | Pressure | | Pres+year | | 40 | |
|  |  | Specific humidity | | Shum+year | | 40 | |
|  |  | Wind speed | | Wind+year | | 40 | |
|  |  | 2m-air temperature | | Temp+year | | 40 | |
|  |  | Precipitation rate | | Prec+year | | 40 | |
| Anthropogenic | | Population count | | Tpop+year | | 6 | |
|  |  | Population density | | Pd+year | | 21 | |
|  |  | Population trend | | Pt | | 1 | |
|  |  | Nighttime lights | | NTL+year | | 21 | |
|  |  | Human footprint | | FP+year | | 2 | |
|  |  | Gross domestic product | | GDP+year | | 5 | |
|  |  | Land cover/use | | Land+year | | 30 | |
| Soils | | pH | | ph+depth  (0-5cm, ph05)  (5-15cm, ph515)  (15-30cm, ph1530)  (30-60cm, ph3060)  (60-100cm, ph60100)  (100-200cm, ph100200) | | 6 | |
|  |  | Soil organic carbon | | soc+depth | | 6 | |
|  |  | Total nitrogen | | tn+depth | | 6 | |
|  |  | Total phosphorus | | tp+depth | | 6 | |
|  |  | Total potassium | | tk+depth | | 6 | |
|  |  | Cation exchange capacity | | cec+depth | | 6 | |
|  |  | Coarse fragment content | | cf+depth | | 6 | |
|  |  | Sand | | btsnd+depth | | 6 | |
|  |  | Silt | | btslt+depth | | 6 | |
|  |  | Clay contents | | btcly+depth | | 6 | |
|  |  | Bulk density | | bd+depth | | 6 | |
|  |  | Thickness | | thickness | | 1 | |
|  |  | Soil moisture | | SM+year+month | | 198 | |
| **CODCLAB_Level 2**  Lake-basin scale characteristics assigned to the basins are stored in shapefiles associated with lake-basin polygons. | | | | | | | |
| Category | Attributes | | Colums | | Unit of values | | Count |
| Hydrology | Lake extent | | LWA+occurrence | | km^2^ | | 2 |
|  | Lake volume | | Lake_vol | | km^3^ | | 1 |
|  | Residence time | | Res_time | | days | | 1 |
|  | Watershed area | | Basin_area | | km^2^ | | 1 |
|  | Supply coefficient | | SC | | dimensionless | | 1 |
| Topography | Elevation | | Elevation | | m | | 1 |
|  | Terrain slope | | Slope | | degrees | | 1 |
|  | Relief amplitude | | RA | | m | | 1 |
| Climate | Temperature | | Tem+year | | 0.1℃ | | 36 |
|  | Precipitation | | Pre+year | | 0.1mm | | 36 |
|  | Evapotranspiration | | Eva+year | | mm | | 36 |
|  | Pressure | | Pres+year | | pa | | 40 |
|  | Specific humidity | | Shum+year | | kg/kg | | 40 |
|  | Wind speed | | Wind+year | | m/s | | 40 |
|  | 2m-air temperature | | Temp+year | | k | | 40 |
|  | Precipitation rate | | Prec+year | | mm/hr | | 40 |
|  | Seasonal SPEI | | SPEI+season | | dimensionless | | 4 |
| Anthropogenic | Population count | | Tpop+year | | number/km^2^ | | 6 |
|  | Population density | | Pd+year | | number/km^2^ | | 21 |
|  | Population trend | | Pt | | count/km^2^/5yrs | | 1 |
|  | Nighttime lights | | NTL+year | | w/cm^2^/sr | | 21 |
|  | Human footprint | | FP+year | | dimensionless | | 2 |
|  | Gross domestic product | | GDP+year | | 10^4^Yuan/km^2^ | | 5 |
|  | Land cover/use | | Type (cp et.al,)+year | | km^2^ | | 279 |
| Soils | Soil property | | Attri (12)+depth (6) | | Attri unit+cm | | 77 |
|  | Soil moisture | | SM+year | | m³/m³ | | 16 |

| **CODCLAB_Level 3**  Tables of all lake-basin attributes associated with the lake-basin ID. | |
| --- | --- |
| Name | Number of sheets |
| Hydr_CODCLAB.xlsx | 5 |
| Topo_CODCLAB.xlsx | 3 |
| Clim_CODCLAB.xlsx | 9 |
| Anth_CODCLAB.xlsx | 7 |
| Soil_CODCLAB.xlsx | 13 |
| **CODCLAB_sub-basins**  CODCLAB of sub-basins for five large lakes (ID39, ID110, ID131, ID330 and ID533). | |
| Level1 | Tiff raster layers |
| Level2 | Shapefiles |
| Level3 | Tables |


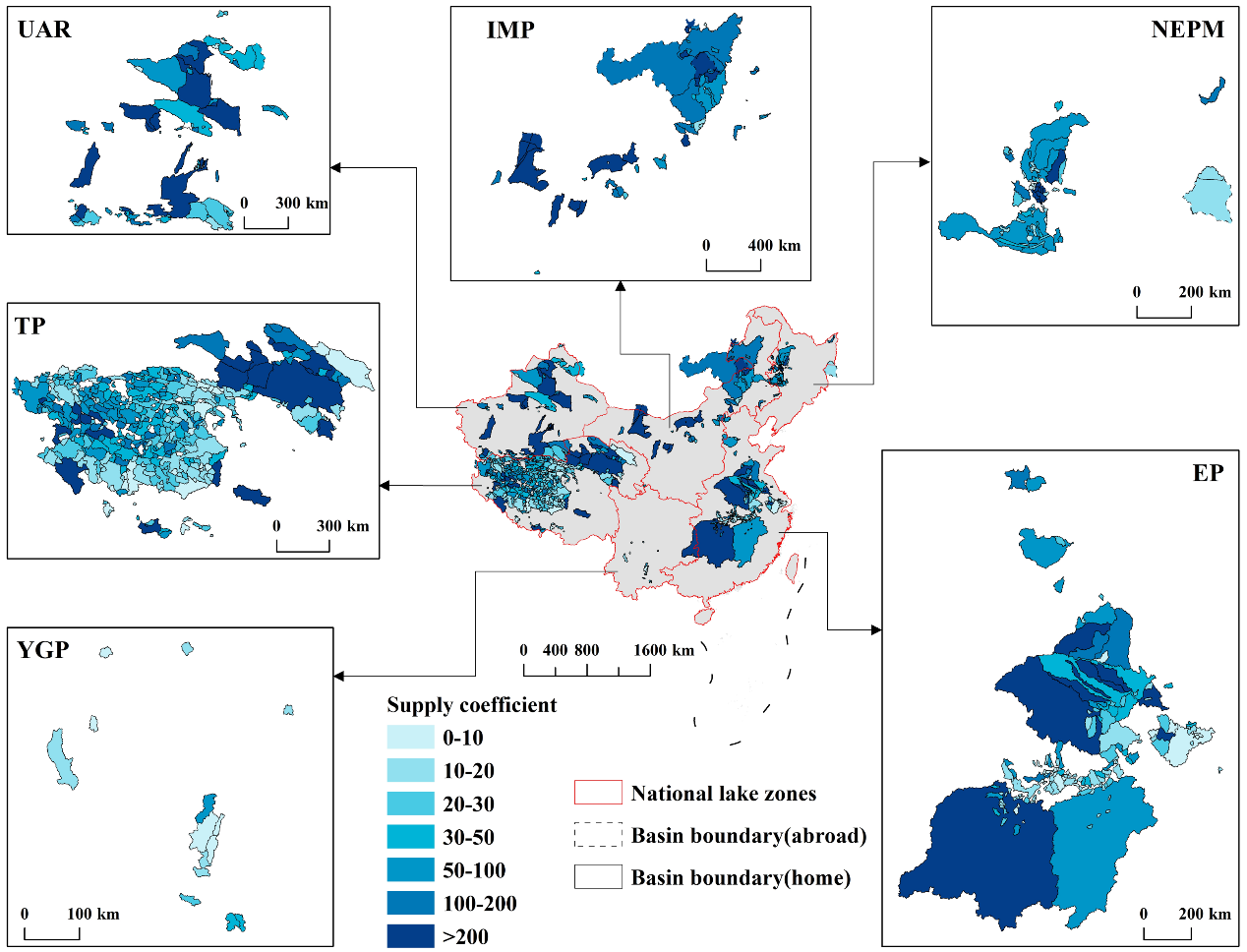


Fig. S2 Supply coefficient of lakes with water occurrence above 75% (represent permanent water).


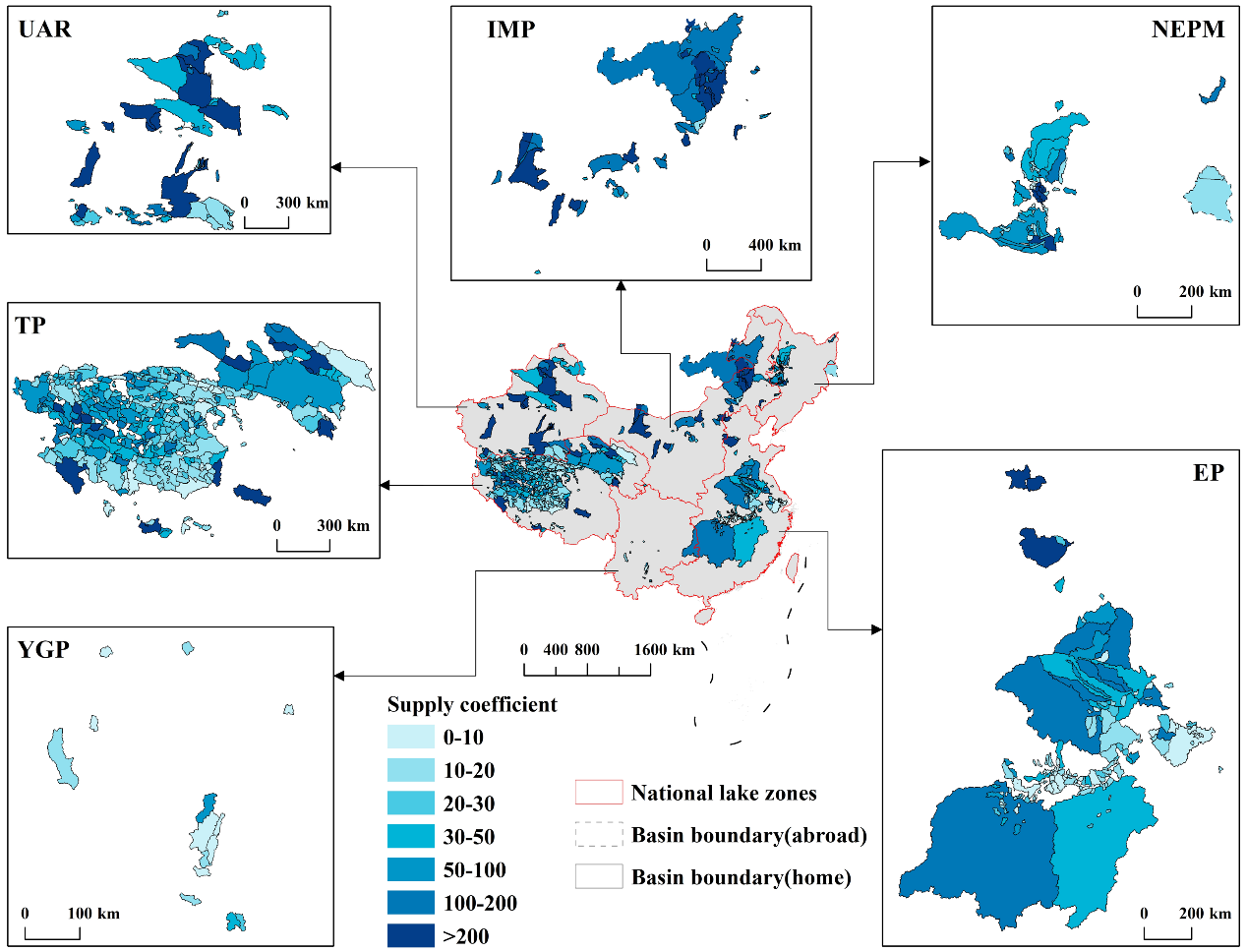


Fig. S3 Supply coefficient of lakes with water occurrence above 25% (represent seasonal water).
